# Immunotherapy-Related Adverse Events and Clinical Outcomes in Adult Solid-Tumor Patients Admitted to an Onco-Hospitalist Medicine Service

**DOI:** 10.3390/cancers17030403

**Published:** 2025-01-25

**Authors:** Cesar Simbaqueba Clavijo, Orhue Odaro, Ayush Gandhi, Kwame Koom-Dadzie, Arine Musaelyan, Kodwo Dickson, Rosalie Chua, Viraj Bhise, Magdelene Amoateng, Sophy Tomy, Daniel Leal Alviarez, Ei Moe Phyu, Ivana Bogdanich, Clark Andersen, Ajay Sheshadri, Nicolas L. Palaskas, Josiah Halm, Joanna Manzano

**Affiliations:** 1 Department of Hospital Medicine, The University of Texas MD Anderson Cancer Center, Houston, TX 77030, USA; 2 Pharmacy Clinical Programs, The University of Texas MD Anderson Cancer Center, Houston, TX 77030, USA; 3 Department of Biostatistics, The University of Texas MD Anderson Cancer Center, Houston, TX 77030, USA; 4 Department of Pulmonary Medicine, The University of Texas MD Anderson Cancer Center, Houston, TX 77030, USA; 5 Department of Cardiology, The University of Texas MD Anderson Cancer Center, Houston, TX 77030, USA

**Keywords:** immune checkpoint inhibitor, immunotherapy, immune-related adverse event, immunotherapy–organ toxicity, hospitalist, hospital medicine

## Abstract

**Background/Objectives**: Few studies have focused on patients with immune-related adverse events (irAEs) after immune checkpoint inhibitor (ICI) treatment who were cared for primarily by hospitalists. The objective of our study was to describe the patterns and outcomes of adult solid-tumor cancer patients admitted to our onco-hospital medicine service. **Methods**: We retrospectively reviewed patients with solid tumors who received ICIs and were admitted to our service in 2021–2022 with an irAE and compared them to a control group (IOTOX vs. NO IOTOX, respectively). The primary outcome was the patterns of irAEs requiring hospitalization; secondary outcomes included 30-day emergency room visit, readmission, and 30-day mortality. **Results**: There were 144 patients in the IOTOX group and 286 controls. The most common tumor type was lung and thoracic malignancies (62, 43.1%). The most common ICI causing the irAEs was pembrolizumab (66, 45.8%). The most common irAEs were pneumonitis (49, 34%), colitis (28, 19.4%), hepatitis (18, 12.5%), and myocarditis (16, 11.1%). Of the 144 patients, eight (6%) died from the hospitalization irAE. Fifteen (15.6%) had an ER visit within 30 days due to the same irAE, and thirteen (13.7%) were readmitted. Survival at 30 days after discharge did not differ significantly between groups. **Conclusions**: Despite many patients having severe irAEs and irAEs associated with higher mortality, they generally had a favorable outcome compared to the literature.

## Simple Summary

Toxicity from immunotherapy is not uncommon. Patients with adverse events after immune checkpoint inhibitor treatment are being increasingly cared for by hospital medicine providers. We characterized the patterns and outcomes of these hospitalizations by conducting a retrospective study of patients cared for by onco-hospitalists in our tertiary center. We found that despite many patients having advanced cancer stage and high-grade adverse events, the majority had favorable outcomes similar to those of admitted patients without immunotoxicity. Furthermore, our patients had lower mortality rates for typically high mortality adverse events such as myocarditis.

## 1. Introduction

The advent of immune checkpoint inhibitors (ICIs) has revolutionized cancer care in the last decade. ICIs have offered a new line of therapy in place of or in combination with conventional chemotherapy agents, improving survival and in effect giving new hope to patients with cancer [1,2]. Compared to traditional targeted therapies, the expanding utility of ICIs lies in their unique mechanism of action, which involves inhibition of natural inhibitory checkpoints for the immune system which exist to prevent autoimmunity [3]. Inhibition of these checkpoints leads to disinhibition of immune cells, which in turn can create new immune responses to a broad range of cancers, leading to cell death [3].

The ICI categories approved by the US Food and Drug Administration (FDA) are PD-1 inhibitors (nivolumab, pembrolizumab, and cemiplimab), PD-L1 inhibitors (atezolizumab, durvalumab, and avelumab), and a CTLA-4 inhibitor (ipilimumab). Most recently, relatlimab, an LAG3 inhibitor, was approved for use in melanoma in combination with nivolumab [4,5,6]. Ipilimumab, pembrolizumab, and nivolumab were the first FDA-approved ICIs; ipilimumab led the way with approval for melanoma in 2011 [4,6]. Since then, ICIs have become a mainstay in the treatment of many other solid tumors, including colorectal, gastric, hepatocellular, and bladder cancers, and have had a lasting impact on patient outcomes [7]. For example, 5-year survival rates for certain tumors, such as melanoma and non-small cell lung cancer, have dramatically improved with the introduction of nivolumab [8].

However, these benefits of ICIs are not without their risks for complications. The literature abounds with studies of the short- and long-term effects of immune-related adverse events (irAEs) associated with ICIs [9,10,11,12,13]. These agents are known to cause immunologic phenomena in multiple organ systems, with dermatologic, lung, gastrointestinal, and endocrine irAEs being most common and the degree of severity depending on tumor type, dose, and use of combination ICI therapy [14,15,16,17]. A significant number of patients treated with ICIs also experience chronic irAEs, particularly affecting the rheumatologic and endocrine systems [18]. Additionally, some irAEs, such as myocarditis and encephalitis, while rare, are associated with high mortality [19,20,21,22].

Patients hospitalized with irAEs typically have higher Common Terminology Criteria for Adverse Events (CTCAE)-grade toxicities and experience higher mortality compared to hospitalized patients who do not receive ICIs [9,10]. A multidisciplinary approach with general internists and internal medicine specialists is crucial to the care of patients with irAEs [23,24]. With the rise of hospital medicine as a subspecialty in the US, hospitalists have become increasingly involved in the management of patients admitted with cancer- and treatment-related complications. The Society of Hospital Medicine has recognized the expanding role of hospitalists in the care of patients with irAEs and published practice points for the management of toxicity in accordance with American Society of Clinical Oncology guidelines [25].

Our large academic comprehensive cancer center has a dedicated group of oncology hospitalists, or onco-hospitalists, defined as internal-medicine-trained physicians who provide patient care solely in an inpatient cancer care setting. Our onco-hospitalist group cares for approximately 40% of admitted solid-tumor patients and is the primary team for the care of cancer patients admitted with irAEs. Our patients include those with thoracic, head and neck, breast, gastrointestinal, and endocrine malignancies. Several studies have presented types and patterns of irAEs in hospitalized patients receiving ICIs, some reporting on specific cancer types or organ systems, and some on specific ICIs [12,14,26,27,28,29,30,31]. Other studies have described outcome variations in patients managed in the inpatient setting [12,24,30,32]. However, there is a paucity of data on patients with irAEs cared for primarily by hospitalists, and data on the care of this patient cohort specifically by onco-hospitalists are limited.

To fill this gap, the objectives of this study were to describe the patterns and outcomes of adult solid-tumor cancer patients receiving ICIs admitted to our medical center onco-hospital medicine service with irAEs.

## 2. Materials and Methods

Under an IRB-approved protocol for the Division of Internal Medicine and the Immunotherapy Toxicity Operational Platform, we performed a retrospective study of all adult solid-tumor cancer patients who received ICIs and were admitted with an irAE to our hospital medicine service at The University of Texas MD Anderson Cancer Center between 1 January 2021 and 31 December 2022. We identified this group as our immunotherapy–organ toxicity (IOTOX) group and reviewed each patient’s first hospitalization for an irAE during the study period. In parallel, we identified as a control group adult solid-tumor cancer patients who received ICIs and were hospitalized within the study period for condition(s) other than an irAE. Of these, we randomly chose a control cohort selecting 2 controls per IOTOX case in a 1:2 matching. We identified this cohort as the NO IOTOX group and reviewed their first hospitalization within the study period. 

Patients’ demographics, characteristics, comorbidities, cancer history, treatment, medications, and hospitalization outcomes were collected (Table 1, Table 2 and Table 3). The primary outcome was the patterns of irAEs requiring hospitalization. The secondary outcomes were as follows: irAE types and management, ICU utilization, improvement of irAEs, length of stay, 30-day emergency room (ER) visit or readmission after discharge, inpatient mortality, mortality at 30 days after discharge, resumption of ICI treatment after discharge, and overall survival. 

Initial statistical analyses were performed using descriptive statistics: a *t*-test was used for the analysis of continuous variables for measures of associations between the 2 groups, and the chi-square test was used for the analysis of the categorical variables. To control for baseline differences between the study groups, we used inverse probability of treatment weighting (IPTW). A gradient-boosted model was used to determine the propensity scores from the relation between study group and all baseline variables, and IPTW-average treatment effect (ATE) weights for each patient were determined from that model. Subsequently, IPTW-weighted logistic regressions related each outcome variable to the study group, while adjusting for several covariates, which otherwise remained poorly balanced between the study groups. Odds ratios and P-values were calculated, giving statistical significance to *p* < 0.05. 

IPTW-weighted Kaplan–Meier analyses and graphics were generated. Overall survival was calculated for duration of inpatient hospitalization, time from discharge (first 30 days), and time from admission. 

## 3. Results

### 3.1. Patient Population

Our total patient population was 430 patients: 144 patients (33.5%) who were hospitalized during our study period with an irAE (immunotherapy–organ toxicity group, IOTOX), and 286 controls (66.5%) who were treated with ICIs and hospitalized for reasons other than irAEs (NO IOTOX). The mean ages of the groups were 67.7 ± 12.2 vs. 65.6 ± 12.3 years, respectively. Slightly over half of the patients in the IOTOX group were male (76, 52.8%), and most were white (114, 79.2%). 

Patients in the IOTOX group had a higher incidence of having received a combination of immunotherapies (39 [27.1%] vs. 18 [10.1%]; *p* = 0.0001), and hyperlipidemia (70 [49.0%] vs. 92 [32.6%]; *p* ≤ 0.001) compared to the NO IOTOX group. There was a trend toward a history of heart failure (20 [14.0%] vs. 23 [8.1%]; *p* = 0.063) and acute kidney injury (28 [20.0%] vs. 38 [13.5%]; *p* = 0.09) compared with those in the NO IOTOX group. There were no statistically significant differences in the incidence of hypertension, diabetes, coronary artery disease, or autoimmune disease between the two groups (Table 1). 

### 3.2. Outcomes

#### 3.2.1. irAE Types and Management

Patients in both the IOTOX and NO IOTOX group received a median of 4 doses of immunotherapy. Immunotherapy had to be discontinued or held at the time of admission in 128 (89.5%) vs. 23 (32.9%) patients, respectively (*p* ≤ 0.0001).

**Figure 1:**
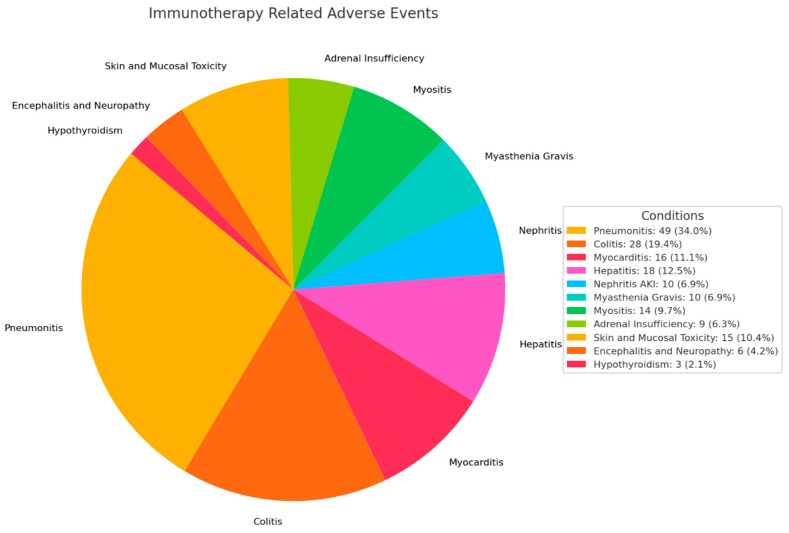
Immunotherapy-related adverse events (irAEs) noted in study population.

The most common irAEs in order of prevalence were pneumonitis (49, 34.0%), colitis (28, 19.4%), hepatitis (18, 12.5%) and myocarditis (16, 11.1%) (Figure 1).

**Figure 2:**
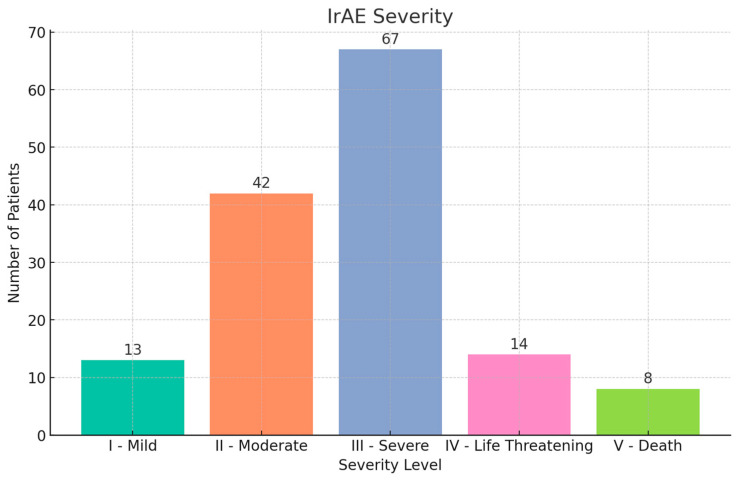
irAE severity by Common Terminology Criteria for Adverse Events (CTCAE) grade.

ICI monotherapies most associated with irAEs were pembrolizumab (66, 45.8%), nivolumab (47, 32.6%), and ipilimumab (31, 21.5%). The combination of an anti-CTLA-4 agent with an anti-PD-1 agent (22, 15.3%) was most associated with an irAE (Table 3). The frequencies of various CTCAE-grade irAEs in order of decreasing severity were as follows: grade 5, 8 (5.6%); grade 4, 14 (9.7%); grade 3, 67 (46.5%); grade 2, 42 (29.2%); grade 1, 13 (9.0% (*p* = 0.054) (Figure 2). 

**Figure 3:**
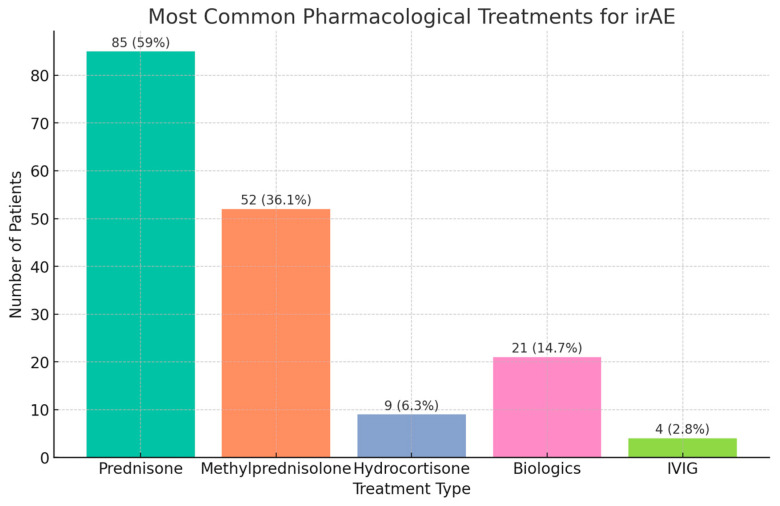
Most common pharmacologic treatments for irAEs.

The pharmacological treatments most employed in the management of irAEs were prednisone (85, 59.0%), methylprednisolone (52, 36.1%), hydrocortisone (9, 6.3%), biologics (21, 14.7%) Rituximab, Infliximab, and Vedolizumab, and intravenous immunoglobin (4, 2.8%). Primarily supportive measures were required in 76 (53.1%) of the patients with irAEs (Figure 3). 

#### 3.2.2. ICU Admission, Clinical Improvement, and Length of Stay

**Figure 4:**
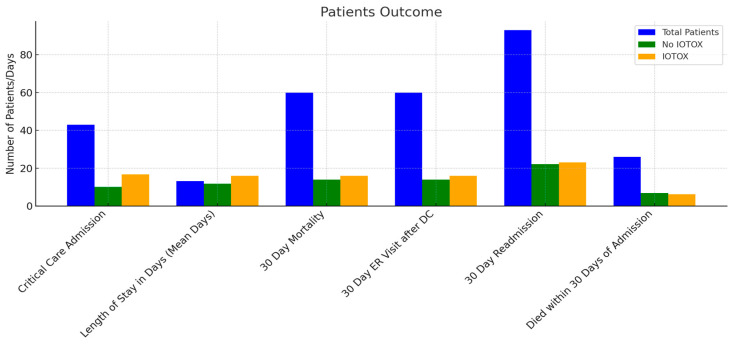
Clinical outcomes of IOTOX vs. NO IOTOX patients.

Patients in the IOTOX group were more likely to require ICU admission during the hospitalization (OR 4.02 [CI 1.58–10.26]; *p* = 0.004). Of the 144 IOTOX patients, 114 (79.2%) were noted to have clinical improvement in the irAE during the hospitalization defined as improvement in symptoms and clinical status. Lengths of stay for the IOTOX vs. NO IOTOX groups were 11.3 days vs. 10.8 days, respectively (Figure 4) (Table 4).

#### 3.2.3. Discharge and Rehospitalization

**Figure 5:**
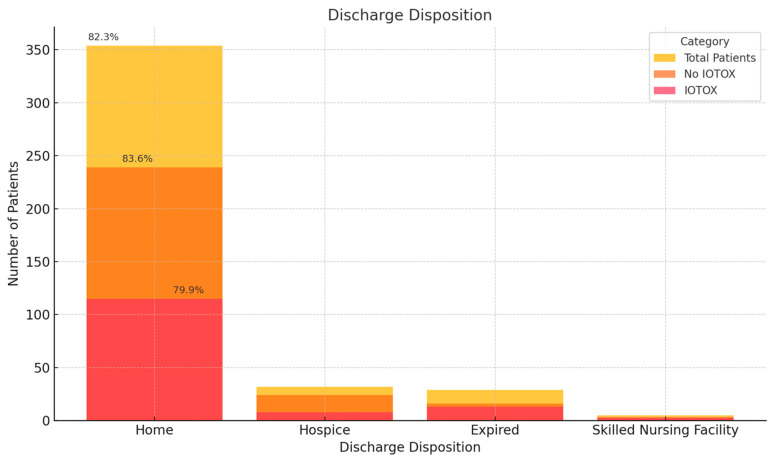
Discharge disposition of IOTOX vs. NO IOTOX patients.

Of the 144 IOTOX patients, 131 were discharged. Discharge dispositions included home, 115 patients (79.9%); hospice, 8 (5.6%); a skilled nursing facility, 3 (2.1%); long-term acute care, 3 (2.1%) (Figure 5). One patient was transferred to another hospital, and one left against medical advice. The 30-day ER visit rates were 38 (26.6%) in the IOTOX group vs. 65 (23.5%) in the NO IOTOX group (*p* = 0.55). Within the IOTOX group, 15 (15.6%) had an ER visit within 30 days for the same irAE.

Patients hospitalized with an irAE were more likely to be readmitted within 30 days of discharge compared to those without an irAE (OR 1.23 [CI 0.58–2.62]; *p* = 0.59), but the difference in risk was not statistically significant. The 30-day readmission rates were 33 (23.1%) in the IOTOX group vs. 60 (21.6%) in the NO IOTOX group (*p* = 0.8). Among patients in the IOTOX group who were later readmitted for the same irAE, 13 (13.7%) patients were readmitted within 30 days. Patients in the IOTOX cohort were more likely to be hospitalized for the same irAE than for other reasons, with an OR of 1.24 (CI 0.56–2.72); *p* = 0.6 (Table 4).

#### 3.2.4. Mortality

Inpatient mortality was 13 (9%) in the IOTOX cohort vs. 16 (5.6%) in the NO IOTOX cohort (*p* = 0.22). The numbers of deaths within 30 days of admission in the IOTOX vs. NO IOTOX groups were 8 (6.3%) vs. 18 (7.1%) (*p* = 1.0). Eight (6.0%) patients in the IOTOX group died of the irAE during the initial hospitalization, five patients from pneumonitis, two from colitis and one from myocarditis. Patients hospitalized with an irAE had a higher all-cause mortality likelihood (OR 1.94 [CI 0.65–5.82]); however, this finding was not statistically significant (*p* = 0.24). Therefore, we found no difference in mortality for the IOTOX group.

#### 3.2.5. Resuming Immunotherapy

Restarting the same ICI treatment as received before the hospitalization within 3 months was seen in 23.8% of the IOTOX group vs. 17.5% of the NO IOTOX group (*p* = 0.45).

#### 3.2.6. Overall Survival

**Figure 6:**
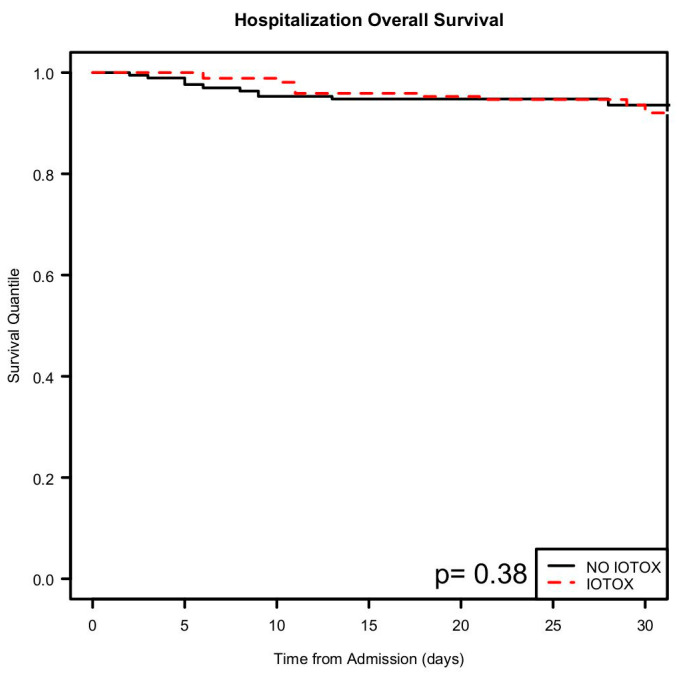
Kaplan–Meier analysis showing hospitalization overall survival.

**Figure 7:**
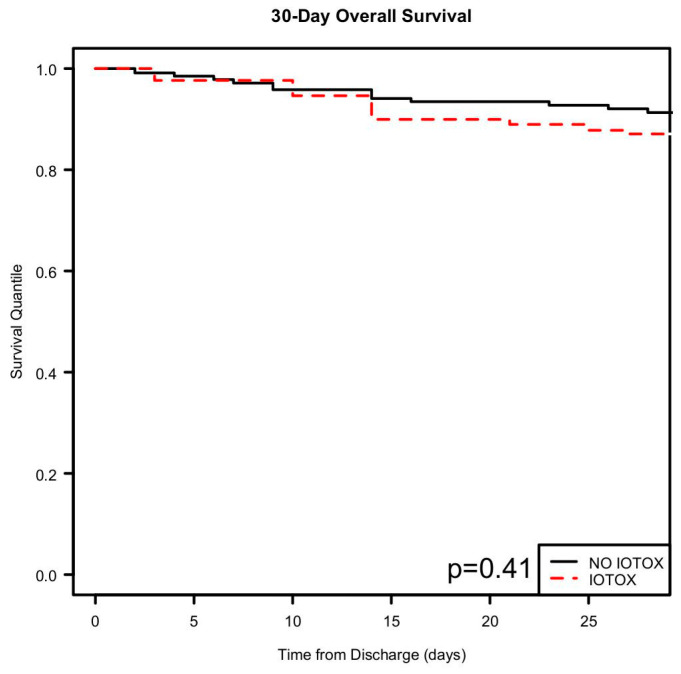
Kaplan–Meier analysis showing 30-day overall survival after discharge.

**Figure 8:**
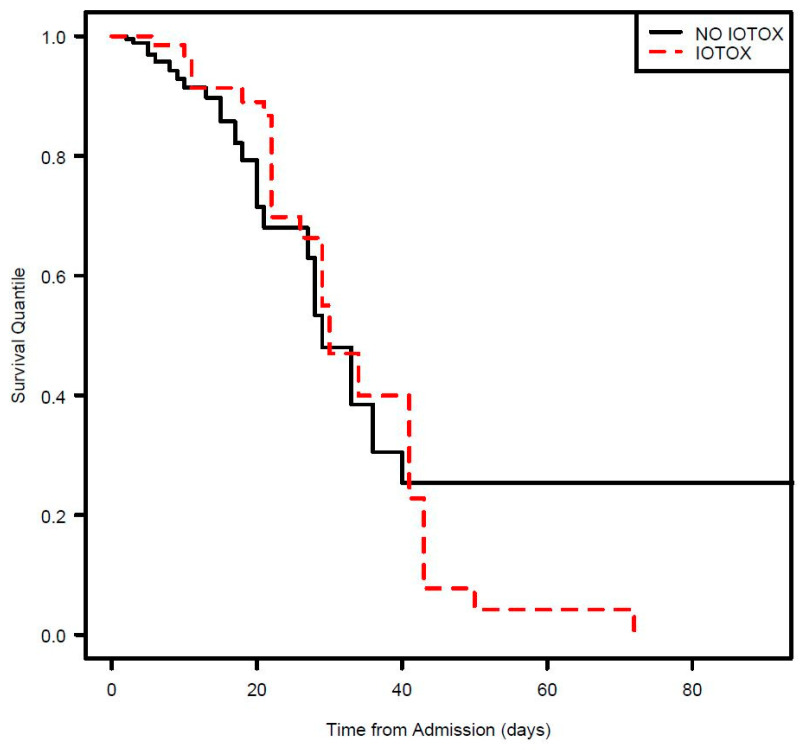
Kaplan–Meier overall survival from time to hospitalization for irAE.

The overall hospitalization inpatient survival was similar for both groups (Figure 6), while the 30 day and beyond overall survival reflected a lower survival for the irAE group (Figure 7 and Figure 8). However, this result was not statistically significant.

## 4. Discussion

Our study describes the patterns and outcomes of solid-tumor patients receiving ICIs who were admitted to our onco-hospital medicine service with irAEs. Our reported outcomes are unique as they highlight the post-hospitalization course, subsequent ER utilization, readmissions for same irAE, and mortality of our patient cohort in a tertiary cancer care center with dedicated onco-hospitalists. 

While both of our groups had similar baseline characteristics, the irAE (IOTOX) group had a higher history of receiving combination immunotherapies consistent with existing literature [33,34]. Kalinich et al. found in a large study looking at predictors of severe irAE requiring hospitalization, that patients who received combination immunotherapy were 2.4 times more likely to require hospitalization for severe immunotherapy-related toxicity [35]. In our study, the most common ICI combination therapy associated with an irAE was an anti-CTLA-4 with an anti-PD-1. The incidence of irAE being most associated with this combination is further supported by existing literature and aligns mechanistically with the points of action of these agents in the immune regulatory cascade [36,37,38,39].

Comparable to another study [9], our IOTOX group had similar lengths of stay (LoS) compared to the NO IOTOX group despite the former being admitted with irAEs. Furthermore, compared to the NO IOTOX group, our IOTOX cohort were more likely to require ICU admission. In healthcare systems, LoS and ICU admission are important metrics for inpatient practitioners as they serve as crucial indicators of the efficiency of healthcare delivery and have significant effects on patient safety, hospital-related complications, quality of care, and healthcare cost [40,41,42,43,44].

We found a higher incidence of pneumonitis and myocarditis in our population than noted in the literature [2], possibly reflecting our status as a referral center, where higher acuity and more complex patients may present for care, as well as increased awareness and protocols for diagnostic workup established at our center. The most common underlying malignancies for our IOTOX patients were lung and thoracic malignancies, as documented in other studies. Pneumonitis was the most common irAE in our patients (34%) and was the most common cause of inpatient-related irAE mortality (62.5%) despite optimal care, highlighting the need for early diagnosis and improved management [45]. Despite dermatological irAEs being one of the most common reported toxicities for patients receiving ICIs [46], their incidence in our study was lower at 10.4%. This may be in part due to close outpatient follow up and early interventions which can lead to better outpatient symptom management and preclude the need for admission. The incidence of the other most common irAEs in our study, colitis and hepatitis, was consistent with literature reports [2].

The overall irAE-associated inpatient mortality rate was low. However, despite the higher incidence of myocarditis in our cohort compared to the literature, the inpatient mortality rates for our patients with myocarditis were lower than in the literature [47,48]. Both the higher incidence and lower inpatient mortality may reflect the expertise at our cancer center in early diagnosis and streamlined management of these patients (thus serving as referral or transfer center), the early recognition of myotoxicity by onco-hospitalists with experience in complex care coordination, and co-management with experts in ICI-induced myocarditis. These positive results highlight the crucial role of a multidisciplinary approach in managing complex cancer patients receiving immunotherapy, as has been reported in similar studies [10]. As has been our experience at our institution, multidisciplinary care teams and boards have shown benefit in patient outcomes especially in complex cases involving significant management recommendations such as discontinuation of ICI therapy, the use of steroids, immunosuppressants and timing of ICI rechallenge [49,50].

Hospitalization overall survival was similar for both groups. However, after 30 days, there was a trend towards decreased survival for the IOTOX group, but we interpret this finding cautiously as it was not statistically significant and is subject to other confounding factors that influence survival, including progression of cancer and the development of other non-irAE medical complications.

As reflected in the literature, most patients in our study who experienced irAEs were undergoing anti-PD-1 monotherapy [9]. Despite a significant number of them experiencing severe-CTCAE-grade toxic effects, the majority demonstrated favorable outcomes, including high rates of improvement, low utilization of the ICU, and low irAE-specific mortality. Compared with prior studies, the lower 30-day irAE ER visit and readmission rate for the same irAE likely reflect an improvement in expertise, recognition, and quality of care over time [51].

The discharge disposition of our patients, which we use as a surrogate for overall debility due to the clinical course, also indicates favorable outcomes for the IOTOX group. Most patients in our study were able to be discharged home despite experiencing higher CTCAE-grade irAEs. After hospitalization, most of the IOTOX patients had the responsible ICI discontinued, and despite the majority experiencing improvement in the irAE, only 28% of them returned to any ICI treatment within 3 months of discharge. Further studies may be needed to better understand this finding.

Limitations of this study include the homogenous nature of the IOTOX cohort (predominantly white or Caucasian), which may limit generalization to other ethnicities and races. Furthermore, melanoma patients, who in the literature constitute a large number of patients receiving ICIs, are not currently cared for by our onco-hospitalist service and thus were not included in the study which can affect the toxicity rates. There is also possible selection bias in which toxicities are admitted, as noted earlier with the example of dermatologic irAEs that are typically managed in the outpatient setting. Finally, this was a single-center study performed in a high-volume referral center. Thus, our patterns and outcomes may differ from smaller hospitals or community-based centers. Nevertheless, our study provides valuable insight into patient outcomes when a dedicated team, such as an onco-hospitalist service, is applied to the management of irAEs.

Future directions include collaborations with other comprehensive cancer centers in a multicenter study to better understand the patterns and outcomes of irAEs in a wider and more varied cohort of patients cared for by onco-hospitalists.

## 5. Conclusions

We found that solid-tumor cancer patients receiving ICIs who developed an irAE requiring hospitalization presented most commonly with pneumonitis, colitis, hepatitis, and myocarditis. The inpatient mortality rates for our patients with myocarditis were lower than reported in the literature. Most patients were undergoing anti-PD-1 therapy. Despite many patients having severe CTCAE-grade toxic effects, they generally had a favorable prognosis, evidenced by high improvement rates, low intensive care utilization, and low irAE-specific mortality. This descriptive analysis provides valuable insights into the patterns and outcomes of IOTOX in this patient population, highlighting the potential for favorable outcomes even in cases of severe toxicities.

## Figures and Tables

**Table 1:** Baseline characteristics: Demographics and comorbidities.

Characteristic	Category	No. of Patients (%)			*p* Value
		Total (N = 430)	NO IOTOX Group (N = 286)	IOTOX Group (N = 144)	
Mean Age (SD), Years		66.3 (12.3)	65.6 (12.3)	67.7 (12.2)	0.08
Gender					
	Female	185 (43%)	117 (40.9%)	68 (47.2%)	0.22
	Male	245 (57%)	169 (59.1%)	76 (52.8%)	
Race/Ethnicity					
	American Indian/ Alaska Native	3 (0.7%)	2 (0.7%)	1 (0.7%)	0.2
	Asian	40 (9.3%)	32 (11.2%)	8 (5.6%)	
	Black or African American	44 (10.3%)	33 (11.6%)	11 (7.6%)	
	White or Caucasian	312 (72.7%)	198 (69.5%)	114 (79.2%)	
	Other/Unknown	30 (7.0%)	20 (7.0%)	10 (6.9%)	
Smoking Status					
	Yes	244 (61.5%)	168 (60.2%)	76 (64.4%)	0.5
	No	153 (38.5%)	111 (39.8%)	42 (35.6%)	
Hypertension					
	Yes	254 (59.5%)	164 (57.7%)	90 (62.9%)	0.35
	No	173 (40.5%)	120 (42.3%)	53 (37.1%)	
Diabetes					
	Yes	113 (26.6%)	72 (25.5%)	41 (28.7%)	0.49
	No	312 (73.4%)	210 (74.5%)	102 (71.3%)	
Chronic Obstructive Pulmonary Disease					
	Yes	79 (18.8%)	51 (18.3%)	28 (19.7%)	0.79
	No	342 (81.2%)	228 (81.7%)	114 (80.3%)	
Autoimmune Disease					
	Yes	19 (4.5%)	12 (4.2%)	7 (4.9%)	0.81
	No	407 (95.5%)	271 (95.8%)	136 (95.1%)	
Hyperlipidemia					
	Yes	162 (38.1%)	92 (32.6%)	70 (49.0%)	0.001
	No	263 (61.9%)	190 (67.4%)	73 (51.0%)	

Most of the patients who developed an irAE had advanced cancer: stage III, 16 (11.1%); stage IV, 108 (75%). The most common primary malignancies were thoracic malignancies (43.1%), followed by gastrointestinal (25.7%) and head and neck tumors (11.1%) (Table 2).

**Table 2:** Baseline characteristics: Cancer status.

Characteristic	Category	No. of Patients (%)			*p* Value
		Total (N = 430)	No IOTOX Group (N = 286)	IOTOX Group (N = 144)	
Tumor Type					
	Brain	2 (0.5%)	1 (0.3%)	1 (0.7%)	1.0
	Head and Neck	59 (13.7%)	43 (15%)	16 (11.1%)	0.3
	Thyroid and Endocrine	21 (4.9%)	13 (4.5%)	8 (5.6%)	0.64
	Lung and Thorax	146 (34.0%)	84 (29.4%)	62 (43.1%)	0.005
	Breast	29 (6.7%)	25 (8.7%)	4 (2.8%)	0.024
	Gastrointestinal	145 (33.7%)	108 (37.8%)	37 (25.7%)	0.013
	Gynecologic	4 (0.9%)	2 (0.7%)	2 (1.4%)	0.6
	Genitourinary	15 (3.5%)	8 (2.8%)	7 (4.9%)	0.28
	Skin	16 (3.7%)	5 (1.7%)	11 (7.6%)	0.005
	Sarcoma	1 (0.2%)	1 (0.3%)	0 (0%)	1.0
	Other	11 (2.6%)	6 (2.1%)	5 (3.5%)	0.52
Stage of Malignancy					
	I	11 (2.6%)	5 (1.7%)	6 (4.2%)	0.24
	II	17 (4.0%)	12 (4.2%)	5 (3.5%)	
	III	46 (10.7%)	30 (10.5%)	16 (11.1%)	
	IV	339 (78.8%)	231 (80.8%)	108 (75.0%)	
	Unknown	17 (4.0%)	8 (2.8%)	9 (6.3%)	
Line of Therapy					
	1st	197 (45.8%)	143 (50.0%)	54 (37.5%)	0.062
	2nd	145 (33.7%)	93 (32.5%)	52 (36.1%)	
	3rd	73 (17.0%)	42 (14.7%)	31 (21.5%)	
	Unknown	15 (3.5%)	8 (2.8%)	7 (4.9%)	

**Table 3:** Baseline characteristics: ICI therapies received.

ICI	Category	No. of Patients (%)			*p* Value
		Total (N = 430)	No IOTOX Group (N = 286)	IOTOX Group (N = 144)	
ICI Type					
	Anti–PD-1	249 (57.9%)	155 (54.2%)	94 (65.3%)	0.03
	Anti–CTLA-4	11 (2.6%)	0 (0%)	11 (7.6%)	<0.0001
	Anti–PD-L1	72 (16.7%)	50 (17.5%)	22 (15.3%)	0.59
	Anti–PD-1/Anti–PD-L1	20 (4.7%)	15 (5.2%)	5 (3.5%)	0.48
	Anti–CTLA-4 with Anti-PD-1	37 (8.6%)	15 (5.2%)	22 (15.3%)	0.0008
	Other ICI	29 (6.7%)	28 (9.8%)	1 (0.7%)	0.0001
Specific ICI					
	Pembrolizumab	196 (45.6%)	130 (45.5%)	66 (45.8%)	1
	Nivolumab	108 (25.1%)	61 (21.3%)	47 (32.6%)	0.013
	Atezolizumab	72 (16.7%)	60 (21.0%)	12 (8.3%)	0.0009
	Ipilimumab	51 (11.9%)	20 (7.0%)	31 (21.5%)	<0.0001
	Durvalumab	14 (3.3%)	1 (0.3%)	13 (9.0%)	<0.0001

**Table 4:** IPTW-weighted logistic regression model.

Patient Outcome	Estimate	Standard Error	Odds Ration NO IOTOX/IOTOX (95% CI)
ICU Admission	1.39	0.48	4.02 (1.58, 10.26)
Died within 30 days of Admission	0.66	0.56	1.94 (0.65, 5.82)
If died, was this patient found to have progression of disease during recent admission	0.44	0.63	1.55 (0.45, 5.33)
30-Day ER Visit	0.21	0.39	1.23 (0.58, 2.62)
30-Day Readmission	0.21	0.40	1.24 (0.56, 2.72)

## Data Availability

The data presented in this study are available on reasonable request from the corresponding author due to institutional legal and research policies.
